# Effectiveness of Apparent Diffusion Coefficient Values in Predicting Pathologic Subtypes and Grade in Non-Small-Cell Lung Cancer

**DOI:** 10.3390/diagnostics14161795

**Published:** 2024-08-16

**Authors:** Hasibe Gokce Cinar, Kemal Bugra Memis, Muhammet Firat Oztepe, Erdem Fatihoglu, Sonay Aydin, Mecit Kantarci

**Affiliations:** 1 Department of Pediatric Radiology, Etlik City Hospital, 06170 Ankara, Turkey; hgecinar@yahoo.com; 2 Department of Radiology, Erzincan Binali Yildirim University, 24100 Erzincan, Turkey; kemalbugramemis@gmail.com (K.B.M.); erdemfatihoglu@erzincan.edu.tr (E.F.); abdulmecit.kantarci@erzincan.edu.tr (M.K.); 3 Department of Radiology, Batman Training and Research Hospital, 72000 Batman, Turkey; firatoztepe92@gmail.com

**Keywords:** non-small-cell lung cancer, diffusion-weighted imaging, ADC value, tumor grade, squamous cell lung cancer, lung adenocarcinoma

## Abstract

Background and Objective: The aim of this study is to evaluate the effectiveness of apparent diffusion coefficient (ADC) values in predicting pathologic subtypes and grade in non-small-cell lung cancer (NSCLC). Materials and Methods: From January 2018 to March 2020, 48 surgically diagnosed NSCLC cases were included in this study. To obtain ADC values, ADC maps were constructed, and a region of interest was put on the tumor. The values were measured three times from different places of the lesion, and the mean value of these measurements was recorded. All MRI scans were evaluated by two radiologists in consensus. Results: A total of 14 cases were squamous cell cancer, 32 cases were adenocarcinoma, and 2 cases were large cell carcinoma. The mean ADC values of adenocarcinoma, squamous cell carcinoma, and large cell cancer were 1.51 ± 0.19 × 10^−3^ mm^2^/s, 1.32 ± 0.15 × 10^−3^ mm^2^/s, and 1.39 ± 0.25 × 10^−3^ mm^2^/s, respectively. There were 11 grade 1, 27 grade 2, and 10 grade 3 NSCLC cases. The mean ADC value was 1.44 ± 0.14 × 10^−3^ mm^2^/s in grade 1 tumors, 1.25 ± 0.10 × 10^−3^ mm^2^/s in grade 2 tumors, and 1.07 ± 0.15 × 10^−3^ mm^2^/s in grade 3 tumors. The cut-off value to discriminate grade 2 from grade 1 tumors was 1.31 ± 0.11 × 10^−3^ mm^2^/s (85% sensitivity, 75% specificity). The cut-off value to discriminate grade 3 from grade 2 tumors was 1.11 ± 0.15 × 10^−3^ mm^2^/s (87% sensitivity, 69% specificity). Conclusions: ADC values can accurately predict NSCLC histopathologic subtypes and tumor grade.

## 1. Introduction

Lung cancer is the most prevalent form of malignant neoplasm and the primary cause of mortality. Based on the latest GLOBOCAN forecasts, there were over 2 million newly diagnosed cases worldwide in 2018. Lung cancer is the next most common type of cancer in males after prostate cancer, with approximately 1.3 million cases. It is the next most common type of cancer in women after breast cancer, with approximately 725,000 cases [1,2,3].

Lung cancer is classified into two main groups, small-cell lung cancer (SCLC) and non-small-cell lung cancer (NSCLC), based on the origin of the cells. NSCLC is undergoing further division [1]. According to the 2021 WHO classification, the most common forms of NSCLC are adenocarcinoma, squamous cell carcinoma (SCC), and neuroendocrine tumors, such as large cell neuroendocrine carcinoma (LCNEC) and carcinoid [4]. The most common histological subtype of lung cancer is adenocarcinoma. SCC accounts for 20% of primary lung cancers and is the second most common subtype in the United States [5].

In thoracic radiology, the diagnosis of pulmonary lesions is typically made based on a detailed analysis of the morphological features of the lesion as visualized on computed tomography (CT) scans. Although certain specific features have been clearly defined to help distinguish between benign and malignant lesions, the use of CT alone as a standalone imaging method can sometimes lead to diagnostic difficulties and uncertainties. Additionally, various pathological conditions in which the normal lung structure is significantly damaged, such as in cases of lung fibrosis, further complicate the evaluation and accurate assessment of the lesion with CT imaging alone. These challenges show that CT imaging alone will not be sufficient for a comprehensive evaluation of pulmonary lesions, and additional imaging modalities will be needed [6].

Multiple therapeutic approaches exist for lung cancer, which differ according to the histological subtype of the tumor. Hence, accurately anticipating the pathological attributes of the tumor is essential in order to select the appropriate therapeutic approach. The literature suggests that the apparent diffusion coefficient (ADC) values obtained from diffusion-weighted magnetic resonance imaging (DWI) can be utilized to showcase the histological subtypes of lung malignancies [5].

Recent studies showed that the ADC may emerge as a candidate biomarker associated with histopathological cancer subtypes and the biological properties of tissues. However, DWI of the lung is problematic due to the biochemical and magnetic properties of lung tissue and the physiological movements caused by the heart and large vessels. These factors make it challenging to standardize ADC values, thus raising doubts about their reliability as a biomarker. With the continuous development of MRI technology, fast DWI methods for the lung have been developed. These advanced DWI methods have significantly minimized the artifacts caused by physiological movements within the lung, enhancing the potential of DWI to provide reliable diagnostic information [7,8].

The rapid growth of MRI procedures, such as echo-planar imaging sequences, multichannel coils, and parallel imaging, has made DWI a practical and efficient tool for detecting and identifying tumors [6,9]. DWI of the lung can be performed using two methods: breath hold scanning and free breath scanning. The breath hold imaging method is advantageous because it does not require a long time; images can be taken in a relatively short period of time. However, this method has some notable disadvantages. These include a decrease in the signal-to-noise ratio at high b values, which can compromise the quality of the images, and a low spatial resolution, which can affect the detail and accuracy of the images obtained. On the other hand, free breath imaging can be performed with either cardiac triggering or respiratory triggering to prevent movement artifacts caused by the patient’s breathing. Cardiac triggering is effective in preventing pulsation artifacts that can occur due to the heart’s motion, but this method typically takes a longer time to complete the imaging process [10]. Respiratory triggering, on the other hand, also plays a significant role in improving the quality of DWI by reducing artifacts that may occur due to respiratory movements, thus providing clearer and more accurate images [11,12]. The 2019 Japanese treatment guidelines recommend the use of MRI for diagnosing lung cancer [13]. DWI has demonstrated considerable promise in distinguishing between malignant and benign pulmonary lesions by using ADC for differential diagnosis in many organs, including the lung, breast, and prostate [9,14]. DWI and ADC mapping have been applied in lung cancer cases in recent years, including the definition of pathological subtypes, tumor grading, establishing the patient’s treatment approach, and predicting treatment response [15]. 

MRI stands out with its better imaging of soft tissue, multiparametric features, and absence of ionizing radiation compared to CT and PET/CT. There is a connection between the functional and metabolic information of imaging methods and the biophysical properties of tissues. DWI identifies microscopic Brownian motions of water in biological tissues. It provides information about diffusion limitations in tissues, reflecting the properties of biological tissues [7,16]. A quantitative assessment of water molecule diffusion in biological tissues may be achieved by measuring ADC values. Malignant tumors exhibit a significantly lower ADC value compared to normal tissues or benign lesions [17]. In a meta-analysis, Wu et al. [18] showed that DWI can effectively distinguish between malignant and benign lung lesions. Moreover, research has shown that the use of functional DWI is superior to CT in evaluating the effectiveness of chemotherapy and/or radiation in lung tumors [19].

In lung cancer, ADC values have the potential to differentiate between benign and malignant lesions. Furthermore, they can differentiate between various histological subtypes of NSCLC. There are some studies that suggest that ADC values may be prognostic biomarkers correlating with tumor grades. Tumors with lower ADC values tend to have higher grades and more aggressive behavior and thus have a poor prognosis [20].

DWI, which is a non-invasive imaging method and ensures valuable knowledge about the tumor microenvironment, is promising in the management of lung cancer. Integrating ADC values with clinical practices could play a valuable role in guiding the diagnosis and management of lung cancer.

Recently, radiomics has emerged as a non-invasive method for extracting high-dimensional data from radiological images, supplementing the standard methods used in lung cancer diagnosis. This approach offers insights into the cellular and molecular properties of the tissue in addition to the visible characteristics of the tumors, providing a more comprehensive understanding. Radiomics features are valuable for distinguishing between different types of tumors, determining prognosis, and guiding treatment through objective and quantitative data [21].

While there is a substantial body of research exploring the importance of DWI in distinguishing between benign and malignant lung masses, there is a scarcity of studies addressing the diagnostic use of ADC values. The purpose of this study was to assess the diagnostic effectiveness of ADC values in distinguishing between different tumor grades and pathological subtypes in NSCLC.

## 2. Materials and Methods

The local ethical committee authorized the study protocol for evaluating the use of DWI in lung cancer patients (ethics committee no. Ebyü-kaek-2021-3-2543.32465.11). Because of this investigation’s methodology, the informed consent requirement was waived.

### 2.1. Study Design and Population

In this retrospective cross-sectional investigation, we analyzed MRI scans to establish the correlation between ADC levels and histopathologic subtypes, as well as the tumor grade, in patients with NSCLC.

The criteria for inclusion were as follows: (1) easily accessible chest MRI scans in the medical records of our hospital, spanning from January 2018 to March 2020; (2) patients who had not received any prior treatment; (3) a complete chest MRI examination with DWI data and no missing MRI sequences; and (4) patients who were operated on in our hospital and whose pathology results were obtained after the MRI scan. (1) Patients receiving neoadjuvant chemoradiotherapy, (2) patients who underwent MRI examination but were not operated on in our hospital, (3) patients with a history of lung operation, (4) patients without DWI images in MRI examination, and (5) unusable chest MRI scans (with motion artifacts, etc.) were excluded.

### 2.2. MRI Protocol

All MRI examinations were conducted using a 1.5-Tesla MRI scanner (MAGNETOM Aera; Siemens Healthineers, Erlangen, Germany) equipped with two six-channel body phased-array coils positioned in the front. The MR images were generated using a coronal T1-weighted spin-echo sequence, an axial fat-saturated T2-weighted sequence, and coronal and axial T2-weighted fast spin-echo sequences. The axial plane was used for the DWI using a single-shot, echo-planar imaging procedure. The DWI series included the following parameters: In a respiratory-triggered scan, the thickness of each slice was 6 mm. The TR/TE/flip angle values were in the range of 3000–4500/65/90. The b value was between 0 and 800 s/mm^2^. The field of vision was 350 mm, and the matrix size was 128 × 128. The ADC maps were automatically created from each DW image using the MR system software (syngo^®^ MR E11, Siemens Healthineers, Erlangen, Germany).

### 2.3. Measurements and Interpretation

The cross-sectional images were examined via PACS. Two radiologists, one with 9 years of expertise and the other with 11 years of experience, collaboratively evaluated all MRI images. The ADC maps were automatically created using the MR system software, and a region of interest (ROI) was positioned on the tumor to acquire ADC data. Values were calculated at three different points on the lesions. Calculations from cystic–necrotic parts were avoided, and the mean values of these measurements were recorded. A two-dimensional (2D) round ROI area was standardized to 1 cm^2^. The obtained ADC values were compared according to both the tumor grade and histopathological subtypes of the NSCLC.

### 2.4. Statistical Analysis

IBM SPSS Statistics for Windows version 22.0 (IBM Corp., Armonk, NY, USA) was used for all statistical analyses. The normal distribution of the data was tested with the Kolmogorov–Smirnov test. Continuous parameters with normal distribution were stated as the mean ± standard deviation. Categorical data were stated as frequencies (n) and percentages (%). The mean ADC values of the numerical variables in the pathological subtypes and various tumor grade subgroups were compared using a one-way ANOVA test. The effectiveness and success of the diagnostic test were defined by an ROC curve analysis and shown as the positive/negative predictive value, sensitivity, and specificity. Predictive values were calculated using the Youden index. A two-tailed value of *p* < 0.05 was considered statistically significant.

## 3. Results

During our retrospective analysis, we reviewed chest MRI scans of 72 individuals that were conducted within the designated time frame. A total of 10 patients were eliminated from the research due to the unavailability of pathology data, while 12 patients were omitted because DWI pictures were not acquired. Two patients were eliminated because of inadequate imaging quality. 

Totally, 48 pathologically diagnosed NSCLC cases after surgery were included in the study. Of these, 29 (60%) were male and 19 (40%) were female. The mean age was 66.12 ± 8.3 years (range: 47 to 88). 

**Figure 1:**
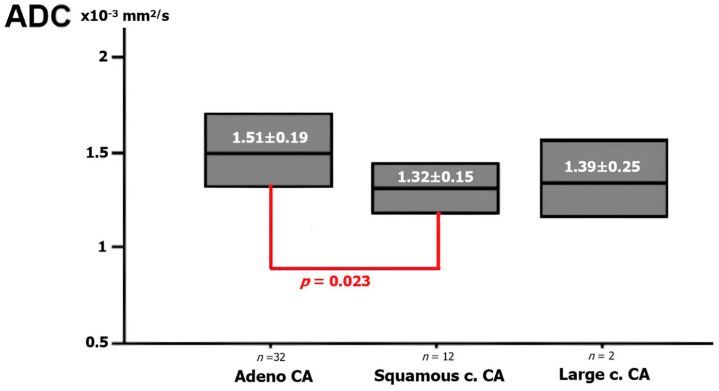
The ADC value for pathologic cell types of NSCLC. The graph shows the ADC values according to each NSCLC pathological subtype. There is a statistically significant difference between adenocarcinoma and SCC, but no significant difference was found between adenocarcinoma and LHH and between SCC and LHH.

A total of 14 cases were squamous cell cancer, 32 cases were adenocarcinoma, and 2 cases were large cell carcinoma. Figure 1 shows the mean ADC values for NSCLC pathologic cell types. The mean ADC value of adenocarcinoma (1.51 ± 0.19 × 10^−3^ mm^2^/s) was substantially greater than that of squamous cell (1.32 ± 0.15 × 10^−3^ mm^2^/s) carcinoma (*p* = 0.023). The mean ADC value of large cell cancer was 1.39 ± 0.25 × 10^−3^ mm^2^/s. No significant difference was found between squamous cell cancer and large cell cancer or between the mean ADC values for adenocarcinoma and large cell cancer (*p* = 0.073 and *p* = 0.061).

**Figure 2:**
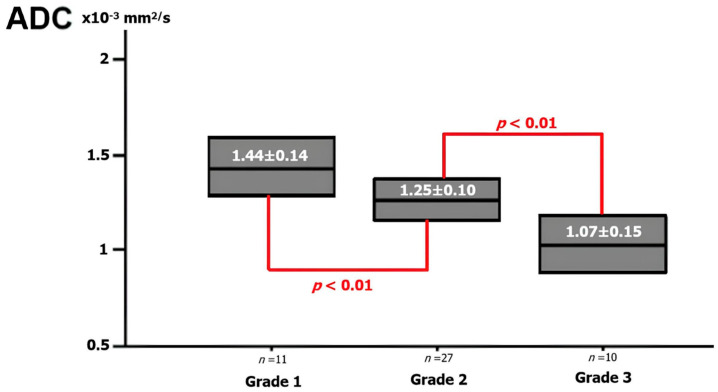
The ADC value for the pathologic grade of NSCLC. The graph shows ADC values according to each NSCLC pathological grade. There is a statistically significant difference between grade 1 and grade 2 and between grade 2 and grade 3 tumors.

There were 11 grade 1, 27 grade 2, and 10 grade 3 NSCLC cases. Figure 2 shows the mean ADC values for tumor grades. The mean ADC value was 1.44 ± 0.14 × 10^−3^ mm^2^/s in grade 1 tumors, 1.25 ± 0.10 × 10^−3^ mm^2^/s in grade 2 tumors, and 1.07 ± 0.15 × 10^−3^ mm^2^/s in grade 3 tumors. We found a significant negative correlation between tumor grade and ADC values (*p* < 0.01). 

**Figure 3:**
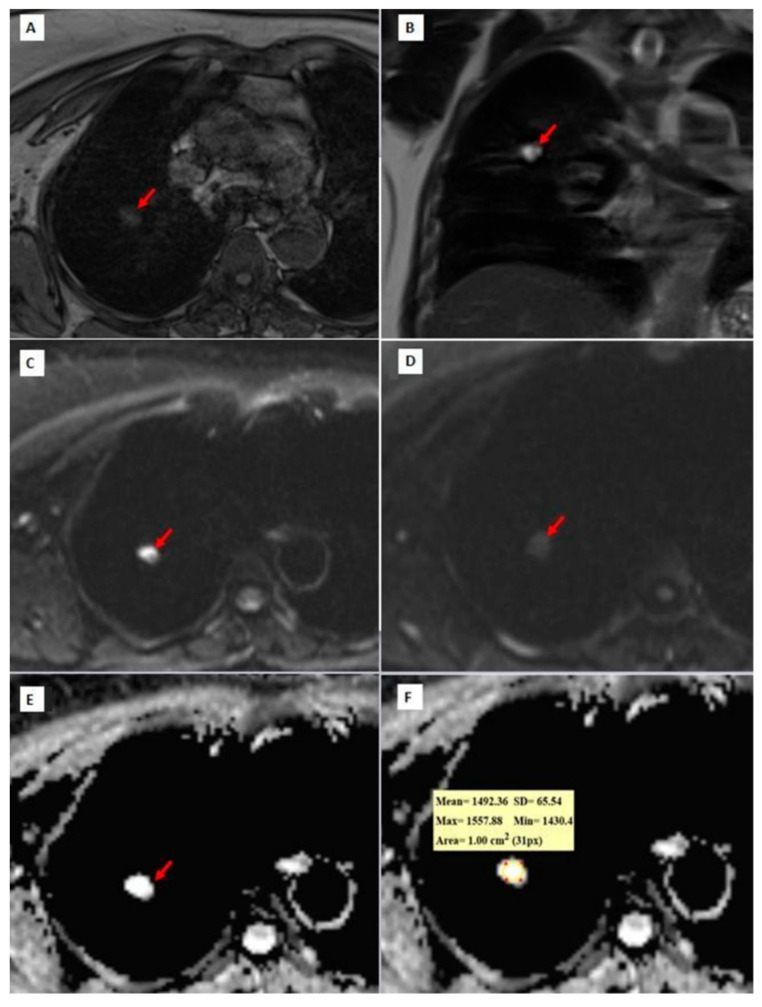
A 72-year-old patient diagnosed with grade 1 adenocarcinoma. (**A**) An axial T1W image showing a hypo-isointense nodule with lobulated contour at the right upper lobe (red arrow). (**B**) A coronal T2W MRI image showing a hyperintense right upper lobe lung nodule (red arrow); (**C**,**D**) b = 0 and b = 800 DW images are shown, respectively. In the b = 0 DW image, there is a hyperintense nodule in the upper lobe of the right lung. In the b = 800 DW images, it is seen that the nodule has lost its signal significantly. (**E**,**F**) In the ADC map, the mean ADC value is measured as 1492.36 × 10^−6^ mm^2^/s within the lesion.

**Figure 4:**
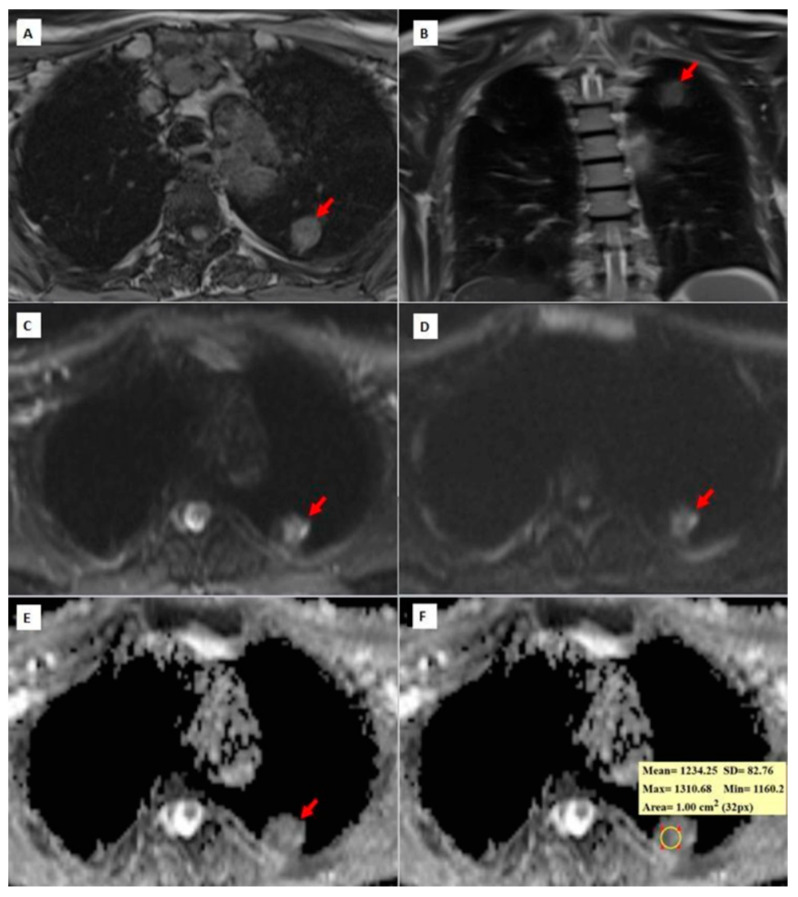
A 62-year-old patient diagnosed with grade 2 squamous cell carcinoma. (**A**) An axial T1W image showing an isointense, round-shaped lung nodule with a slightly spiculated extension towards the pleura in the upper lobe of the left lung. (**B**) A coronal T2W MRI image showing an isointense left upper lobe lung nodule (red arrow); (**C**,**D**) b = 0 and b = 800 DW images, respectively. In the b = 0 DW images, the nodule has a heterogeneous internal structure and there is a more hyperintense area on the left side of the lesion. In the b = 800 DW images, it is seen that the signal of the lesion decreases slightly and the left side remains more hyperintense than the lesion. (**E**,**F**) ADC maps showing a hypointense nodule and a mean ADC value of 1234.25 × 10^−6^ mm^2^/s. While placing the ROI for measurement on the ADC maps, the asymmetric signal area on the left side of the lesion was excluded in the DW images in order to prevent the DW parameters from being affected by non-tumor areas, such as necrosis, abscess, etc., within the tumor.

**Figure 5:**
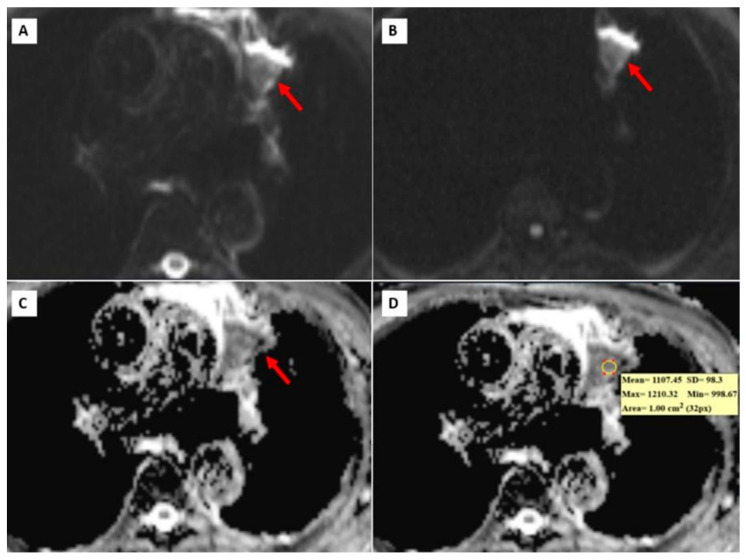
A 68-year-old patient with grade 3 squamous cell carcinoma. (**A**,**B**) b = 0 and b = 800 DW images, respectively. A heterogeneous left upper lobe lung mass (red arrows). (**C**,**D**). ADC maps showing a heterogeneous mass and a mean ADC value of 1107.45 × 10^−6^ mm^2^/s.

Figure 3, Figure 4 and Figure 5 show thorax MRI images of patients with tumors in the grade 1, 2, and 3 categories, respectively.

The cut-off value to discriminate grade 2 from 1 tumors was 1.31 ± 0.11 × 10^−3^ mm^2^/s (85% sensitivity, 75% specificity). The cut-off value to discriminate grade 3 from 2 tumors was 1.11 ± 0.15 × 10^−3^ mm^2^/s (87% sensitivity, 69% specificity).

## 4. Discussion

Lung cancer is one of the most important causes of cancer-related deaths worldwide, as stated in numerous studies and supported by statistical data [1]. The prognosis and treatment outcomes of lung cancer vary significantly depending on the histological subtypes and the stage at which the cancer is diagnosed. Therefore, early and accurate determination of these histological subtypes is of critical importance for effective treatment planning and management. Despite advances in imaging technologies and molecular diagnostic methods, there are still some challenges in the diagnosis and prognosis of lung cancer due to the heterogeneous structure and complex behavior of the tumor, which can vary greatly among patients. 

Fluoro-2-deoxy-glucose positron emission tomography/computed tomography (FDG-PET/CT) is a widely used imaging technique in lung cancer staging and in distinguishing between benign and malignant nodules [22]. The maximum standardized uptake value (SUVmax) provided by FDG-PET/CT scans offers critical information about the aggressiveness of the tumor. However, there are instances where this method can yield false negative or false positive results, which pose a challenge to its reliability [17]. Additionally, the high radiation dose and significant cost associated with FDG-PET/CT scans constitute considerable limitations of this imaging method.

Recent studies showed that the ADC may emerge as a candidate biomarker associated with histopathological cancer subtypes and the biological properties of tissues. However, DWI of the lung is problematic due to the biochemical and magnetic properties of lung tissue and the physiological movements caused by the heart and large vessels. These factors make it challenging to standardize ADC values, thus raising doubts about its reliability as a biomarker. With the continuous development of MRI technology, fast DWI methods for the lung have been developed. These advanced DWI images have significantly minimized the artifacts caused by physiological movements within the lung, enhancing the potential of DWI to provide reliable diagnostic information [7,8].

The rapid growth of MRI procedures, such as echo-planar imaging sequence, multichannel coils, and parallel imaging, has made DWI a practical and efficient tool for detecting and identifying tumors [6,9]. DWI of the lung can be performed using two methods: breath hold scanning and free breath scanning. The breath hold imaging method is advantageous because it does not require a long time; images can be taken in a relatively short period. However, this method has some notable disadvantages. These include a decrease in the signal-to-noise ratio at high b values, which can compromise the quality of the images, and a low spatial resolution, which can affect the detail and accuracy of the images obtained. On the other hand, free breath imaging can be performed with either cardiac triggering or respiratory triggering to prevent movement artifacts caused by the patient’s breathing. Cardiac triggering is effective in preventing pulsation artifacts that can occur due to the heart’s motion, but this method typically takes a longer time to complete the imaging process [10]. Respiratory triggering, on the other hand, also plays a significant role in improving the quality of DWI by reducing artifacts that may occur due to respiratory movements, thus providing clearer and more accurate images [11,12]. The 2019 Japanese treatment guidelines recommended the use of MRI for diagnosing lung cancer [13]. DWI has demonstrated considerable promise in distinguishing between malignant and benign pulmonary lesions by using ADC for differential diagnosis in many organs, including the lung, breast, and prostate [9,14]. DWI and ADC mapping have been applied in lung cancer cases in recent years, including the definition of pathological subtypes, tumor grading, and establishing the patient’s treatment approach and predicting treatment response [15]. 

DWI is performed with at least two b values. There is no standardized b value in the lung. Increasing the b value increases the sensitivity for detecting diffusion restriction, but this time, image distortion occurs. This affects the image quality more, especially in an organ such as the lung, which is prone to artifacts in MR imaging. In our study, we used two b values: 0 and 800 s/mm^2^ [23].

In line with the findings of Usuda et al.’s study [19] involving 226 patients, our current investigation reveals that adenocarcinoma exhibits a much higher ADC value compared to squamous cell carcinoma or large cell carcinoma. Adenocarcinomas include a glandular architecture and produce mucin. This increases the extracellular space and water diffusion. Thus, adenocarcinomas tend to have higher ADC values [12]. In contrast, squamous cell carcinomas and large cell carcinomas have higher cellularity and denser stromal components with lower ADC values [17]. Unfortunately, according to the data collected by Shen et al. [6] in a meta-analysis, a correlation between the ADC values and histological types of lung carcinoma has been suggested; this suggests that ADC measurements may be helpful to distinguish the subtypes of NSCLC. Regrettably, the combined results indicate that there was a convergence of ADC values among the different histological categories of lung cancer. Consequently, ADC measurements were unable to discern between the subtypes of NSCLC. There are some factors, such as keratinization, stratification, and cellular atypias for the classification of lung cancer. These factors play a critical role in defining the histological category of the tumor as they provide detailed information about the cellular architecture and differentiation status of the cancer cells. Furthermore, necrosis, abscess, and other lesions can significantly affect ADC measurements. Abscess with necrosis has low ADC values because it blocks mobility due to its high cellularity and viscosity [24]. These factors can obscure the true diffusion characteristics of tumor tissue. During this study, we found that DWI scanning could reveal histological necrosis and mucinous regions in lung cancer that can affect the ADC values. For accurate ADC measurements, we avoid placing the ROI in these areas and instead perform it from three different points within each lesion. This helps us avoid misleading necrosis and mucinous regions on ADC values and provides a more reliable measurement of tumor diffusion characteristics. Furthermore, similar to the literature, our investigation found a relationship between ADC values and pathological structures [6,19,25,26]. 

The pathological type and grade are prognostic factors for lung cancer that can be used in treatment [20]. The ADC value has been used for prognostic factors in some cancer types, including gliomas, prostate cancer, and breast cancer [27,28,29]. Wang et al. evaluate the effectiveness of fractional anisotropy and ADC values in distinguishing grade 2 and 3 brain gliomas. They reported that the cut-off value of the minimum ADC value was 0.895 × 10^−3^ mm^2^/s and that the sensitivity and specificity were 81.0% and 89.1%, respectively [27]. In their study, Yan et al. investigated the relationship between ADC values in distinguishing low- and intermediate-risk prostate cancer and the Gleason score, which is a prognostic factor for prostate cancer; they reported that the invasiveness of prostate cancer was correlated with low ADC values, and the cut-off values for the discrimination of low- and intermediate-risk prostate cancer were 0.703 × 10^−3^ mm^2^/s for the minimum ADC and 0.927 × 10^−3^ mm^2^/s for the mean ADC, while the sensitivity and specificity were determined to be 85% and 85% for the minimum ADC and 89% and 86% for the mean ADC, respectively [28]. 

In our study, we investigate the relationship between the ADC value and the prognosis of lung cancer. Our findings indicate that there is a substantial drop in the mean ADC values as the grade of tumors increases in patients with NSCLC. Patients were categorized into grade 1, grade 2, or grade 3 depending on the grades of their tumors. The study conducted by Li et al. on the relationship between ADC values and tumor differentiation degree and pathological subtypes in lung cancer reported that the cut-off value of the mean ADC values in differentiating between well- and moderately-differentiated NSCLC and poorly differentiated NSCLC was 1059.5 × 10^−6^ mm^2^/s, and the sensitivity and specificity were reported as 88% and 76%, respectively. There was a significant difference in the ADC values between small-cell carcinoma and other NSCLC subtypes, but there was no significant difference between SCC and adenocarcinoma [7]. In Kumar et al.’s study that investigated the effectiveness of DW imaging in distinguishing between malignant and benign pulmonary nodules, the mean ADC cut-off value was reported as 1209 × 10^−6^ mm^2^/s, and the sensitivity and specificity values were reported as % 65.2 and % 87.5, respectively [23]. In the ADC histogram analysis conducted by Tsuchiya et al. [15], it is seen that the ADC values corresponding to the 50% percentile of grades 1, 2, and 3 in NSCLC are 1.37, 1.18, and 1.09, respectively. The existing literature contains research that provides evidence of a negative association between tumor grade and ADC levels, as seen in [20,30,31]. The finding that a decreased ADC value is correlated with an elevated pathological tumor grade in NSCLC provides compelling evidence that the ADC value has the potential to be utilized for prognosis evaluation.

This study has some limitations. Significantly, this study conducted a retrospective single-center investigation, which may limit the generalizability of the results. The sample size was relatively small, which could affect the robustness of the statistics. Further research with larger populations is needed to improve the statistical power of the findings. Moreover, we acquired the DWI using a 1.5-T MR scanner with two different b-values in our investigation. The use of a more potent magnetic field, such as a 3.0-T scanner, could potentially enhance the quality of the images and provide more detailed information. More external validation from multiple centers would help to determine the reproducibility and reliability of the results across different clinical settings. The retrospective nature of this study also introduces potential biases which could influence the findings. Additionally, there is a lack of integration of ADC values with other imaging modalities and molecular biomarkers that can affect the diagnostic accuracy in lung cancer. 

## 5. Conclusions

ADC levels are highly helpful in predicting the tumor grade and histopathologic subtypes of NSCLC. The mean ADC value of adenocarcinoma (1.51 ± 0.19 × 10^−3^ mm^2^/s) was greater than that of squamous cell (1.32 ± 0.15 × 10^−3^ mm^2^/s) carcinoma (*p* = 0.023). ADC levels are also helpful in predicting the tumor grade. The mean ADC value is 1.31 ± 0.11 × 10^−3^ mm^2^/s for discriminating grade 2 from grade 1 tumors, and the mean ADC value is 1.11 ± 0.15 × 10^−3^ mm^2^/s for discriminating grade 3 from grade 2 tumors. These values can provide information about the tumor cellularity and structure in addition to traditional histopathological methods. However, additional validation is required for the use of ADC values in clinical practice. Prospective studies are needed in larger populations in which ADC values are compared with other conventional methods to determine the effectiveness of ADC values in the discrimination and categorization of different types of lung lesions. Additionally, the integration of ADC values with other imaging and molecular biomarkers may increase diagnostic accuracy in lung cancer.

## Data Availability

Patient data are not available to the public due to privacy and confidentiality issues.
